# Oral administration of *Bifidobacterium breve* B-3 modifies metabolic functions in adults with obese tendencies in a randomised controlled trial

**DOI:** 10.1017/jns.2015.5

**Published:** 2015-05-04

**Authors:** Jun-ichi Minami, Shizuki Kondo, Naotake Yanagisawa, Toshitaka Odamaki, Jin-zhong Xiao, Fumiaki Abe, Shigeru Nakajima, Yukie Hamamoto, Sanae Saitoh, Taeko Shimoda

**Affiliations:** 1 Food Science and Technology Institute, Morinaga Milk Industry Co. Ltd, Zama, Kanagawa, Japan; 2 Nutritional Science Institute, Morinaga Milk Industry Co. Ltd, Zama, Kanagawa, Japan; 3 NAKAJIMA Medical Clinic, Yokosuka, Kanagawa, Japan; 4 Division of Healthcare Graduate School, Tokyo Healthcare University, Setagaya, Tokyo, Japan

**Keywords:** *Bifidobacterium*, Metabolic syndrome, Obesity, Randomised controlled trials, DIO, diet-induced obesity, γ-GTP, γ-glutamyltranspeptidase, HbA1c, glycated Hb, hCRP, high-sensitivity C-reactive protein, LPS, lipopolysaccharide

## Abstract

Accumulating evidence suggests an association between gut microbiota and the development of obesity, raising the possibility of probiotic administration as a therapeutic approach. *Bifidobacterium breve* B-3 was found to exhibit an anti-obesity effect on high-fat diet-induced obesity mice. In the present study, a randomised, double-blind, placebo-controlled trial was conducted to evaluate the effect of the consumption of *B. breve* B-3 on body compositions and blood parameters in adults with a tendency for obesity. After a 4-week run-in period, the participants were randomised to receive either placebo or a B-3 capsule (approximately 5 × 10^10^ colony-forming units of B-3/d) daily for 12 weeks. A significantly lowered fat mass was observed in the B-3 group compared with the placebo group at week 12. Improvements were observed for some blood parameters related to liver functions and inflammation, such as γ-glutamyltranspeptidase and high-sensitivity C-reactive protein. Significant correlations were found between the changed values of some blood parameters and the changed fat mass in the B-3 group. These results suggest the beneficial potential of *B. breve* B-3 in improving metabolic disorders.

Obesity is becoming a global epidemic and a major contributor to the increased incidence of serious chronic diseases, such as type 2 diabetes, CVD, hepatic and skeletal muscle insulin resistance, and certain types of cancer^(^^1^^)^. The main cause of obesity is an imbalance between energy intake and expenditure. However, there is a growing body of evidence that not only energy intake and expenditure habits explain the increasing prevalence of obesity; environmental factors contribute as well^(^^2^^)^.

Even within monozygotic twins, some individuals are more prone to weight gain, suggesting that factors other than the human genome are involved in the development of obesity^(^^3^^)^. The gut microbiota is an example of an environmental factor that may affect the development of obesity. Recent evidence suggests that the gut microbiota has a supporting function in regulating energy balance, fat storage, neurohormonal functions and immune systems^(^^4^^–^^7^^)^. Accordingly, recent studies have suggested that manipulating the composition of the microbial ecosystem in the gut may be a novel approach in the treatment of obesity. Such treatment might consist of altering the composition of the microbial communities of an obese individual by administering beneficial micro-organisms, commonly known as probiotics^(^^2^^)^.

In the context of obesity, several studies have reported that a low number of *Bifidobacterium* spp. is correlated with the development of obesity and/or diabetes^(^^8^^–^^10^^)^. Furthermore, Cani *et al.*^(^^11^^,^^12^^)^ demonstrated that diet-induced obesity (DIO) (high-fat–low-carbohydrate diet) in mice markedly affects the gut microbial community, in which the levels of *Bifidobacterium* spp. were significantly reduced, in agreement with observations in human subjects. Ilmonen *et al.*^(^^13^^)^ applied *Lactobacillus rhamnosus* together with *Bifidobacterium lactis* Bb12 to prevent obesity during and after pregnancy, which resulted in reduced body weight. Mikirova *et al.*^(^^14^^)^ found that a probiotic combination of both *L. acidophilus* and *B. bifidum* reduced body weight among fifty-three obese individuals. A double-blind randomised controlled trial by Kadooka *et al.*^(^^15^^)^ demonstrated that the administration of the microbial strain *Lactobacillus gasseri* SBT2055 reduced adiposity and body weight. The authors also demonstrated the reproducible effect of SBT2055 to lower the visceral fat mass, BMI, and waist and hip circumferences by a randomised controlled trial using fermented milk with a lower concentration of *L. gasseri* SBT2055.

Along with the evidence of the therapeutic effects of probiotic bacteria against obesity, some mechanisms of action have been proposed, such as an effect on appetite regulation, host metabolism, the inhibition of lipid absorption, the maintenance of intestinal homeostasis and integrity, and low-grade inflammation^(^^16^^–^^18^^)^. However, the effects of these probiotic bacteria are believed to be strain dependent, and the underlying mechanism remains unclear^(^^19^^)^. To establish a therapeutic approach by which the administration of probiotic bacteria can prevent obesity, further research for each bacterial strain is necessary.

We previously reported that the administration of a probiotic strain *B. breve* B-3 in a mouse model of DIO reduced body-weight gain and visceral fat deposits in a dose-dependent manner and improved the serum levels of total cholesterol, glucose and insulin^(^^20^^)^. However, the effect of this strain on human subjects has not yet been investigated.

Here, we conducted a randomised, double-blind, parallel-group comparative study to examine whether the consumption of a capsule containing live *B. breve* B-3 effects body composition and blood parameters in adults with a tendency for obesity.

## Subjects and methods

### Trial design

The present study was designed as a randomised, double-blind, parallel-group comparative study. The protocol was in accordance with the Declaration of Helsinki. All of the participants provided written informed consent. All of the study protocols were approved and controlled by the Ethics Committee of Tokyo Healthcare University (Tokyo Japan). The study was performed from January to June 2012 at the Nakajima medical clinic in Kanagawa Prefecture, Japan.

### Participants

**Fig. 1.**
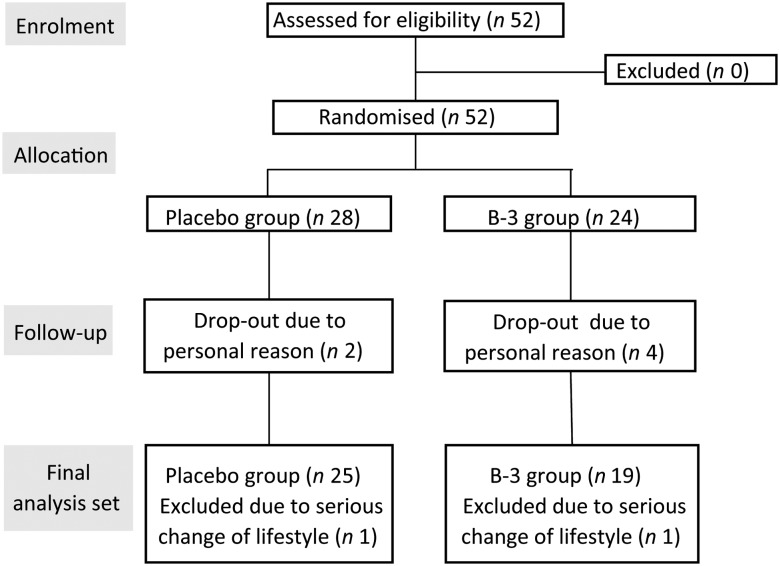
Participant flowchart. B-3, *Bifidobacterium breve* B-3.

A total of fifty-two adult volunteers were recruited based on their BMI (ranging from 24 to 30 kg/m^2^) and age (ranging from 40 to 69 years) at the Nakajima medical clinic. Most of the participants were under clinical practice for diabetes including nutrition education and medication. The exclusion criteria included regular ingestion of diet foods or supplements for lowering body fat or cholesterol (more than twice per week), ingestion of products with probiotic bacteria that could affect the microbiota as an intestinal regulator, a history of severe diseases of the liver, kidney, heart, lung, digestive organs, blood, endocrine system or metabolic pathway, and the presence of a food allergy or drug allergy. A flowchart of participants is shown in Fig. 1.

### Procedure

The study period consisted of a 4-week run-in period, followed by a 12-week ingestion period. Initially, during the run-in period, the participants were instructed to complete a questionnaire of body measurements, lifestyle habits (intake of yogurt, smoking and alcohol drinking), disorders, allergies and regular medications. Each subject made a daily record of their alcohol drinking, exercise and activities, usage of drugs, defecation and physical condition. For the body composition and blood test, the baseline measurement took place at the end of the 4-week run-in period, which was regarded as week 0. After the run-in period, the participants were stratified by sex and BMI (above 27 or less than 27 kg/m^2^) and randomly divided into two groups according to computer-generated permuted-block randomisation at Tokyo Healthcare University. Each group was then randomised to receive three capsules of placebo capsule or B-3 capsule daily for 12 weeks. During the study, the participants were advised not to change their regular lifestyles, including their diet, exercise and regular medications. Then, the subjects initiated the intake of B-3 or the placebo capsule and visited the clinic to receive physical measurements, blood sampling and a medical interview every 4 weeks, i.e. at 4, 8 and 12 weeks after the initiation of capsule intake.

### Test samples

The capsules of B-3 contained lyophilised powder of *B. breve* B-3, a strain originating from a healthy infant, and had mainly maize starch as the carrier in an acid-protective gelatin capsule. The placebo capsule only included an internal matrix (mainly maize starch). We confirmed that the B-3 capsules contained approximately 5 × 10^10^ colony-forming units per three capsules by microbial colony count using reinforced clostridial agar (Oxoid) before the clinical trial. The B-3 and placebo capsules appeared and tasted identical.

### Outcomes

Changes in body composition (body weight, BMI, muscle mass, fat mass, fat percentage, waist:hip ratio) and blood parameters (blood lipids, blood glucose, liver function, inflammatory parameters) at 12 weeks were the primary and secondary outcomes, respectively. Body weight, muscle mass and body fat (amount and percentage) were measured by the bioelectrical impedance method using the medical-grade measurement device InBody3.0 (Takumi). BMI was calculated as body weight (kg)/height (m^2^). The waist:hip ratio was calculated based on the waist circumference and hip circumference. Blood analysis was performed at Health Science Research Institute Co., Ltd (Tokyo, Japan).

### Statistical analysis

SAS software version 9.3 (SAS Institute, Inc.) was used for statistical analyses. Variables that did not follow a normal distribution were analysed after natural log transformation. For clarity, the values are presented according to the original scale in the tables. The baseline characteristics were compared with a Fisher's exact test for categorical variables and a two-sample *t* test for continuous variables. For the primary and secondary outcomes, a comparison at 12 weeks was performed between the experimental groups using ANCOVA adjusted for the baseline (week 0). The within-group change at each time point (weeks 4, 8 and 12) from the baseline (week 0) was analysed using a one-sample *t* test. The correlation between the changing values in the fat mass and those of the other parameters between 0 and 12 weeks were analysed using Pearson's correlation, followed by standardising each variable (*z* score). A *P* value <0·05 was considered statistically significant.

## Results

### Baseline characteristics of participants

Of the participants, four in the B-3 group and two in the placebo group withdrew from the intervention due to personal reasons. One individual in each group reported a serious change of lifestyle (being on a diet by dietary restriction) during the test period; therefore, these participants were removed from the analysis. Thus, the final datasets for the present study were nineteen for the B-3 group and twenty-five for the placebo group (Fig. 1). The baseline values of the subject demographics, physical characteristics, prescribed drug usage (for diabetes mellitus, hyperlipidaemia and hypertension) and metabolic markers (TAG, fasting blood sugar and HDL-cholesterol) did not differ significantly between the B-3 and placebo groups (Table 1). Compliance was high for sample ingestion: 98·4 (sem 2·6) and 102·5 (sem 2·2) % in the B-3 and placebo groups, respectively.

**Table 1.** Baseline characteristics of the subjects (Mean values with their standard errors or number or percentage of participants)

Group…	Placebo		*Bifidobacterium breve* B-3		
	Mean	sem	Mean	sem	P*
Sex (*n*)	25		19		0·535
Male	11		6		
Female	14		13		
Age (years)	61·9	1·9	58·9	2·0	0·302
Body weight (kg)	71·2	2·3	68·9	2·7	0·506
BMI (kg/m^2^)	27·7	0·5	27·1	0·6	0·461
Muscle mass (kg)	45·1	2·0	44·8	2·1	0·908
Fat mass (kg)	23·5	1·0	21·5	1·0	0·187
Fat percentage (%)	33·3	1·3	31·3	1·0	0·276
Prescribed drug use (%)					
For diabetes mellitus	72		79		0·734
For hyperlipidaemia	52		68		0·358
For hypertension	36		42		0·760
Metabolic markers (%)					
TAG ≥ 1·7 mmol/l†	60		42		0·361
Fasting blood sugar ≥ 6·1 mmol/l†	84		79		0·709
HDL-cholesterol < 1·0 mmol/l†	4		5		1·000

* *P* values of Fisher's exact test for categorical data and *P* values of two-sample *t* tests for continuous data are shown.

† Proportion of participants with a baseline level higher than the borderline for domestic criteria of the metabolic syndrome in Japan.

### Effect on body composition

Table 2 shows the transition of each body composition during the intervention. As a primary outcome, the changes in body composition at 12 weeks were analysed by ANCOVA. A significantly lowered fat mass was observed in the B-3 group compared with the placebo group. A tendency for an inter-group difference was observed for the fat percentage, but there was no significant difference for the other parameters between the two groups. During the intervention, a significant decrease from the baseline (week 0) was observed for body weight (weeks 4 and 12 for the B-3 group; week 4 for the placebo group), BMI (week 4 for both groups), fat mass (weeks 4 and 12 for the B-3 group), fat percentage (week 12 for the B-3 group) and waist:hip ratio (weeks 8 and 12 for both groups).

**Table 2.** Changes in physical examination values (Mean values with their standard errors)

Group…	Placebo								*Bifidobacterium breve* B-3								
	Week 0		Week 4		Week 8		Week 12		Week 0		Week 4		Week 8		Week 12		
	Mean	sem	Mean	sem	Mean	sem	Mean	sem	Mean	sem	Mean	sem	Mean	sem	Mean	sem	*P*†
Body weight (kg)	71·2	2·3	70·4*	2·3	71·0	2·3	70·8	2·3	68·9	2·7	68·4*	2·6	68·6	2·6	69·1*	2·6	0·493
BMI (kg/m^2^)	27·7	0·5	27·5*	0·6	27·6	0·5	27·8	0·5	27·1	0·6	26·9*	0·6	27·0	0·6	27·2	0·6	0·403
Muscle mass (kg)	45·1	2·0	44·5	1·9	44·9	1·9	44·8	1·9	44·8	2·1	44·8	2·1	44·9	2·1	45·7	2·1	0·376
Fat mass (kg)	23·5	1·0	23·3	1·1	23·3	1·1	23·4	1·0	21·5	1·0	21·0*	1·0	21·0	0·9	20·8**	0·9	0·046
Fat percentage (%)	33·3	1·3	33·3	1·3	33·2	1·3	33·3	1·2	31·3	1·0	30·8	1·1	30·8	1·0	30·3*	1·0	0·066
Waist:hip ratio	0·964	0·0084	0·960	0·0086	0·958*	0·0085	0·963*	0·0076	0·935	0·0091	0·932	0·0084	0·928**	0·0081	0·931**	0·0071	0·174

Mean value within a group was significantly different from that at baseline (week 0): * *P* < 0·05, ** *P* < 0·01 (one-sample *t* test).

† Differences between the placebo and B-3 groups at week 12 were analysed by ANCOVA, adjusted for baseline (week 0).

### Effect of *Bifidobacterium breve* B-3 on blood parameters

Table 3 shows the transition of blood parameters during the intervention. Significant inter-group differences were observed for the values of γ-glutamyltranspeptidase (γ-GTP) (*P* = 0·011) and high-sensitivity C-reactive protein (hCRP) (*P* = 0·039) at week 12. No significant difference was observed for blood lipids (total, LDL- and HDL-cholesterol, TAG) (data not shown). During the intervention, significant increases from the baseline (week 0) were observed for glycated Hb (HbA1c) (weeks 4 and 8 for both groups, week 12 for the placebo group), γ-GTP (week 12 for the placebo group) and total bilirubin (weeks 4 and 8 for the placebo group). Significant decreases from the baseline were observed for insulin (weeks 4 and 8 for the placebo group), alkaline phosphatase (ALP) (week 8 for the B-3 group) and hCRP (week 12 for the B-3 group).

**Table 3.** Changes in blood parameters (Mean values with their standard errors)

Group…	Placebo								*Bifidobacterium breve* B-3								
	Week 0		Week 4		Week 8		Week 12		Week 0		Week 4		Week 8		Week 12		
	Mean	sem	Mean	sem	Mean	sem	Mean	sem	Mean	sem	Mean	sem	Mean	sem	Mean	sem	*P*†
Fasting blood glucose (mmol/l)‡	8·63	0·72	9·03	1·00	8·51	0·88	8·60	0·74	9·88	0·90	10·16	0·83	9·39	0·66	9·83	0·71	0·541
HbA1c (%)	7·0	0·3	7·2*	0·3	7·2*	0·3	7·3*	0·3	7·5	0·3	7·8*	0·3	7·8*	0·3	7·7	0·2	0·249
Glycoalbumin (%)	18·9	0·7	18·8	0·8	18·8	0·7	19·0	0·8	21·0	1·0	20·6	0·8	20·2	0·7	20·3	3·1	0·213
1,5-Anyhdroglucitol (µg/ml)	9·8	1·3	10·3	1·4	10·5	1·4	10·6	1·4	6·0	0·8	5·7	0·8	5·7	0·8	6·6	0·8	0·725
Insulin (µU/ml)‡§	39·9	10·3	31·8*	8·9	29·1*	8·6	39·4	12·7	32·0	6·1	25·5	4·3	25·9	5·4	37·7	8·0	0·096
AST (IU/l)‡	26·4	3·0	26·6	2·5	26·8	2·6	27·2	3·3	29·7	5·4	28·9	4·5	27·9	4·7	29·5	5·9	0·624
ALT (IU/l)‡	28·6	2·6	29·7	3·1	30·2	3·2	30·6	3·1	34·0	5·3	35·1	5·7	34·2	5·7	32·2	4·9	0·156
ALP (IU/l)‡	209·5	12·3	211·9	13·9	203·5	12·5	207·2	12·0	242·7	20·0	241·7	20·1	230·2*	18·0	234·3	18·4	0·704
Lactate dehydrogenase (IU/l)	199·0	7·9	197·1	7·2	197·8	7·8	199·4	8·5	197·4	6·6	197·6	8·3	196·2	8·6	197·2	8·2	0·936
γ-GTP (IU/l)‡	39·0	6·1	39·6	7·0	42·4	8·1	44·0*	8·4	37·0	7·1	41·7	8·1	39·6	8·2	37·8	7·2	0·011
Total bilirubin (µmol/l)‡	8·90	0·51	11·12*	0·68	10·60*	0·68	10·09	0·68	10·43	0·86	10·77	0·86	10·09	0·86	10·43	0·86	0·233
hCRP (mg/l)‡	0·88	0·14	0·90	0·19	0·93	0·18	1·10	0·18	0·98	0·25	1·27	0·32	1·05	0·23	0·90*	0·27	0·039

HbA1c, glycated Hb; AST, aspartate transaminase; ALT, alanine aminotransferase; ALP, alkaline phosphatase; γ-GTP, γ-glutamyltranspeptidase; hCRP, high-sensitivity C-reactive protein.

* Mean value within a group was significantly different from that at baseline (week 0) (*P* < 0·05; one-sample *t* test).

† Differences between the placebo and B-3 groups at week 12 were analysed by ANCOVA, adjusted for baseline (week 0).

‡ Analysis for within-group and intergroup differences was performed after logarithmic transformation of the values.

§ To convert insulin in µU/ml to pmol/l, multiply by 6·945.

### Correlation of changes in body fat mass with blood parameters

**Fig. 2.**
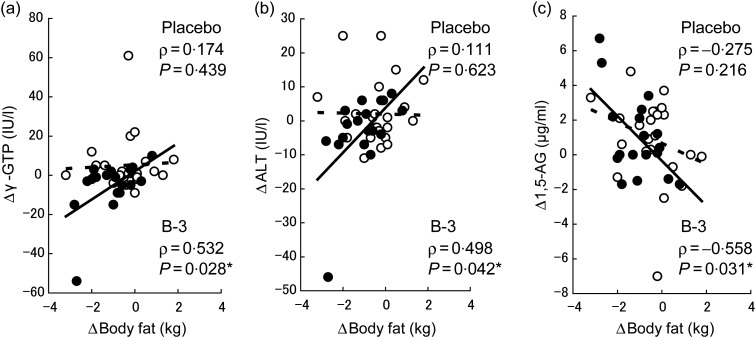
Correlation analysis between changed values at week 12 from week 0 of body fat mass and blood parameters: (a) γ-glutamyltranspeptidase (γ-GTP); (b) alanine aminotransferase (ALT); (c) 1,5-anhydroglucitol (1,5-AG) by Pearson's correlation test. Data shown are correlation coefficients (ρ) with *P* values. * *P* < 0·05. For ALT and γ-GTP, correlation analysis was performed using the data after logarithmic transformation and standardisation. (––––, ●), *Bifidobacterium breve* B-3 (B-3) group; (----, ○), placebo group.

Significant positive correlations were found between the changed values of the body fat mass and those of alanine aminotransferase (ALT) and γ-GTP; negative correlations were found with 1,5-anhydroglucitol in the B-3 group but not in the placebo group (Fig. 2). No significant correlation was found between the changed values of the body fat mass and those of other blood parameters.

## Discussion

We conducted this clinical trial to examine whether the intake of capsules containing a single strain of live *B. breve* B-3 could influence the obese tendencies of adults. In the present study, the B-3 group did not show a significant reduction in body weight compared with the placebo group; however, the fat mass was significantly reduced by the intake of capsules containing probiotic *B. breve* B-3 for 12 weeks. The decreased fat mass was 0·7 kg on average after 12 weeks in the B-3 group. Similar results were observed by Kadooka *et al*.^(^^15^^)^, who showed an average of 0·6 kg reduction in body fat mass after a 12-week ingestion of a *Lactobacillus* strain, *L. gasseri* SBT2055. Although strict diet restriction may induce a dramatic change in fat mass (for example, −12·4 kg after intake of 2090 kJ (500 kcal)/d for 5 weeks), such a serious diet programme may be accompanied by the risk of a rebound effect due to the declined muscle mass and lowered basal metabolism. Also there would be concerns about the difficulty in continuation. There is considerable evidence that visceral fat obesity is a key aetiological factor for the development of metabolic syndromes, and the excess accumulation of visceral fat is primarily involved in metabolic disorders^(^^21^^,^^22^^)^. The present results suggest the potential of *B. breve* B-3 in the prevention of chronic diseases triggered by adiposity in a sustainable way. Since the intervention was only 12 weeks, future study is needed to evaluate the long-term effects of *B. breve* B-3 ingestion.

In the present study, the participants were recruited at a medical clinic, and most of them were under clinical practice for diabetes. Table 1 shows that there was no difference in drug usage between the two groups. In addition, these regular medications were not changed during the intervention. Some participants were prescribed sulfonylureas (56 % in the placebo group and 53 % in the B-3 group) and thiazolidinediones (24 % in the placebo group and 16 % in the B-3 group), diabetes mellitus drugs which have been suggested to affect body weight^(^^23^^)^; however, there was no significant difference between the two groups. Since most of the participants were under clinical practice for diabetes including nutrition education and medication, the values with relation to diabetes such as fasting blood glucose, HbA1c, glycoalbumin and insulin tended to be higher than the normal levels at baseline (Tables 1 and 3). No significant improvement was found in these parameters by B-3 intake as compared with the placebo group during the intervention; however, there was a tendency of improvement for the values of glycoalbumin, a parameter known to reflect the most recent blood glucose status, in the B-3 group (*P* = 0·081 at week 12) (Table 3). In addition, there were significant increases in the levels of HbA1c, a parameter known to reflect blood glucose levels for the previous 2–3 months, during the intervention in the placebo group, but this was disappeared in the B-3 group at week 12 (Table 3). Interestingly, there was a significant negative correlation between the changed values of fat mass and 1,5-anhydroglucitol, a parameter known to closely reflect diabetic control within several days, in the B-3 group but not the placebo group at week 12 (Fig. 2). These results suggest the potential of B-3 in the improvement of diabetes, although further investigation with long-term treatment is needed.

In the present study, we also observed a significant improvement in γ-GTP, which correlated with the reduction of fat mass during the consumption period. The correlation between serum γ-GTP levels and risk of the metabolic syndrome has been indicated in large-scale investigations performed in Korea and Japan^(^^24^^,^^25^^)^. Clinically, evaluations of serum γ-GTP levels are widely used as markers to evaluate the degree of liver injury^(^^26^^,^^27^^)^. In addition, γ-GTP has been used as a marker of hepatobiliary tract function and excessive alcohol consumption^(^^28^^–^^30^^)^. A stronger correlation between serum γ-GTP level and metabolic syndrome risk factors was observed in a non-drinker subgroup compared with all other study subjects^(^^31^^)^. Aller *et al.*^(^^32^^)^ reported that the administration of a tablet of 500 million *Lactobacillus bulgaricus* and *Streptococcus thermophilus* improved liver aminotransferases levels in patients with non-alcoholic fatty liver disease. In a pilot study, short-term oral supplementation with *Bifidobacterium bifidum* and *Lactobacillus plantarum* 8PA3 was associated with an improvement in alcohol-induced liver injury compared with standard therapy alone^(^^33^^)^; however, the effect of probiotics in the improvement of γ-GTP has not be previously evaluated. Although the mechanism of probiotics in the improvement of liver function has not yet been adequately evaluated, the results observed in the present study are in agreement with our previous experimental result showing that feeding DIO mice *B. breve* B-3 led to the regulated gene expression of pathways involved in lipid metabolism, as well as responses to stress in the liver^(^^34^^)^.

Accumulating evidence indicates that obesity is associated with a state of chronic, low-grade inflammation, suggesting that inflammation may be a potential mechanism by which obesity leads to insulin resistance^(^^35^^–^^38^^)^. In the early stages of obesity, adipocytes become hypertrophied in response to overnutrition. With increasing adiposity, adipose tissue releases signals such as monocyte chemoattractant protein-1, causing increased monocyte influx^(^^39^^)^. In the advanced stages of obesity, various types of immune cells, such as macrophages, infiltrate into obese adipose tissue and thus enhance the inflammatory changes through cross-talk with adipose tissue-released NEFA^(^^40^^,^^41^^)^. Lipopolysaccharides (LPS) have been suggested to be involved in the development of metabolic syndromes associated with high fat intake^(^^11^^,^^42^^,^^43^^)^. These reports described that mice fed on a high-fat diet for 2 to 4 weeks exhibited a significant increase in plasma LPS. This increase in plasma LPS induced by a high-fat diet suggests metabolic endotoxaemia, which is considered to trigger the development of obesity, inflammation, insulin resistance, type 2 diabetes and atherosclerosis via activation of the CD14/Toll-like receptor 4 complex by LPS and/or fatty acids. However, supplementation with oligofructose stimulates bifidobacterial growth and lowers the uptake of LPS from the gut lumen^(^^11^^)^. This effect is also correlated with an improved glucose tolerance and insulin sensitivity. Hoarau *et al.*^(^^44^^)^ reported that the supernatant fraction of *B. breve* C50 can induce prolonged dendritic cell survival, with oppositional action on the maturation–apoptosis programme induced by LPS. The *B. breve* supernatant fraction alone was reported to be a poor cytokine inducer but may have immunomodulatory properties^(^^45^^)^. Jeon *et al.*^(^^46^^)^ found that *B. breve* but not *L. casei* induced the development of IL-10-producing Tr1 cells in the large intestine, which demonstrates that *B. breve* prevents intestinal inflammation. In the present study, the 12-week intake of *B. breve* B-3 resulted in significantly decreased hCRP levels compared with the placebo group (Table 3). In our previous study, we observed the down-regulation of the expression of acute-phase proteins, such as *Saa* and *Orm*, by administering *B. breve* B-3 to DIO mice, together with the suppression of body-weight gain and fat accumulation^(^^34^^)^. These results suggest that *B. breve* B-3 may function in suppressing the pro-inflammatory reaction related to obesity.

In summary, the 12-week intake of the probiotic *B. breve* B-3 reduced the fat mass of adult subjects with obese tendencies. Possible improvement was also observed for liver function and the systemic inflammatory reaction by *B. breve* B-3 administration. No serious adverse events were observed by medical interview or measurements of blood parameters. *B. breve* B-3 is capable of producing SCFA, such as acetic acid and lactic acid, and other bioactive components, such as conjugated linoleic acid and other fatty acid metabolites, which are possibly involved in the anti-metabolic syndrome effect. A further large-scale randomised controlled trial is needed to confirm the clinical effect of *B. breve* B-3 on improving metabolic function. A study is underway to identify the active components of *B. breve* B-3 involved in the anti-metabolic syndrome effect.

## References

[1] Haslam DW & James WPT (2005) Obesity. Lancet 366, 1197–1209. 16198769 10.1016/S0140-6736(05)67483-1

[2] Mekkes MC, Weenen TC, Brummer RJ, (2013) The development of probiotic treatment in obesity: a review. Benef Microbes 5, 19–28. 23886977 10.3920/BM2012.0069

[3] Turnbaugh PJ, Hamady M, Yatsunenko T, (2009) A core gut microbiome in obese and lean twins. Nature 457, 480–484. 19043404 10.1038/nature07540 PMC2677729

[4] Zhang H, DiBaise JK, Zuccolo A, (2009) Human gut microbiota in obesity and after gastric bypass. Proc Natl Acad Sci U S A 106, 2365–2370. 19164560 10.1073/pnas.0812600106 PMC2629490

[5] Ley RE, Turnbaugh PJ, Klein S, (2006) Microbial ecology: human gut microbes associated with obesity. Nature 444, 1022–1023. 17183309 10.1038/4441022a

[6] Esteve E, Ricart W & Fernández-Real JM (2011) Gut microbiota interactions with obesity, insulin resistance and type 2 diabetes: did gut microbiote co-evolve with insulin resistance? Curr Opin Clin Nutr Metab Care 14, 483–490. 21681087 10.1097/MCO.0b013e328348c06d

[7] Musso G, Gambino R & Cassader M (2010) Obesity, diabetes, and gut microbiota: the hygiene hypothesis expanded? Diabetes Care 33, 2277–2284. 20876708 10.2337/dc10-0556 PMC2945175

[8] Kalliomäki M, Collado MC, Salminen S, (2008) Early differences in fecal microbiota composition in children may predict overweight. Am J Clin Nutr 87, 534–538. 18326589 10.1093/ajcn/87.3.534

[9] Collado MC, Isolauri E, Laitinen K, (2008) Distinct composition of gut microbiota during pregnancy in overweight and normal-weight women. Am J Clin Nutr 88, 894–899. 18842773 10.1093/ajcn/88.4.894

[10] Wu X, Ma C, Han L, (2010) Molecular characterisation of the faecal microbiota in patients with type II diabetes. Curr Microbiol 61, 69–78. 20087741 10.1007/s00284-010-9582-9

[11] Cani PD, Neyrinck AM, Fava F, (2007) Selective increases of bifidobacteria in gut microflora improve high-fat-diet-induced diabetes in mice through a mechanism associated with endotoxaemia. Diabetologia 50, 2374–2383. 17823788 10.1007/s00125-007-0791-0

[12] Cani PD, Amar J, Iglesias MA, (2007) Metabolic endotoxemia initiates obesity and insulin resistance. Diabetes 56, 1761–1772. 17456850 10.2337/db06-1491

[13] Ilmonen J, Isolauri E, Poussa T, (2011) Impact of dietary counselling and probiotic intervention on maternal anthropometric measurements during and after pregnancy: a randomized placebo-controlled trial. Clin Nutr 30, 156–164. 20970896 10.1016/j.clnu.2010.09.009

[14] Mikirova NA, Casciari JJ, Hunninghake RE, (2011) Effect of weight reduction on cardiovascular risk factors and CD34-positive cells in circulation. Int J Med Sci 8, 445–452. 21850193 10.7150/ijms.8.445 PMC3156990

[15] Kadooka Y, Sato M, Ogawa A, (2013) Effect of *Lactobacillus gasseri* SBT2055 in fermented milk on abdominal adiposity in adults in a randomised controlled trial. Br J Nutr 110, 1696–1703. 23614897 10.1017/S0007114513001037

[16] Sanz Y, Santacruz A & Gauffin P (2010) Gut microbiota in obesity and metabolic disorders. Proc Nutr Soc 69, 434–441. 20540826 10.1017/S0029665110001813

[17] Kadooka Y, Sato M, Imaizumi K, (2010) Regulation of abdominal adiposity by probiotics (*Lactobacillus gasseri* SBT2055) in adults with obese tendencies in a randomized controlled trial. Eur J Clin Nutr 64, 636–643. 20216555 10.1038/ejcn.2010.19

[18] Delzenne NM, Neyrinck AM & Cani PD (2013) Gut microbiota and metabolic disorders: how prebiotic can work? Br J Nutr 109, S81–S85. 23360884 10.1017/S0007114512004047

[19] Yin YN, Yu QF, Fu N, (2010) Effects of four bifidobacteria on obesity in high-fat diet induced rats. World J Gastroenterol 16, 3394–3401. 20632441 10.3748/wjg.v16.i27.3394 PMC2904885

[20] Kondo S, Xiao JZ, Satoh T, (2010) Antiobesity effects of *Bifidobacterium breve* strain B-3 supplementation in a mouse model with high-fat diet-induced obesity. Biosci Biotechnol Biochem 74, 1656–1661. 20699581 10.1271/bbb.100267

[21] Montague CT & O'Rahilly S (2000) The perils of portliness: causes and consequences of visceral adiposity. Diabetes 49, 883–888. 10866038 10.2337/diabetes.49.6.883

[22] Fox CS, Massaro JM, Hoffmann U, (2007) Abdominal visceral and subcutaneous adipose tissue compartments: association with metabolic risk factors in the Framingham Heart Study. Circulation 116, 39–48. 17576866 10.1161/CIRCULATIONAHA.106.675355

[23] Bennett WL, Maruthur NM, Singh S, (2011) Comparative effectiveness and safety of medications for type 2 diabetes: an update including new drugs and 2-drug combinations. Ann Intern Med 154, 602–613. 21403054 10.7326/0003-4819-154-9-201105030-00336 PMC3733115

[24] Nakanishi N, Suzuki K & Tatara K (2004) Serum γ-glutamyltransferase and risk of metabolic syndrome and type 2 diabetes in middle-aged Japanese men. Diabetes Care 27, 1427–1432. 15161799 10.2337/diacare.27.6.1427

[25] Lee JH, Um MH & Park YK (2013) The association of metabolic syndrome and serum γ-glutamyl transpeptidase: a 4-year cohort study of 3,698 Korean male workers. Clin Nutr Res 2, 67–75. 23429457 10.7762/cnr.2013.2.1.67 PMC3572816

[26] Lieber CS (1984) Alcohol and the liver: 1984 update. Hepatology 4, 1243–1260. 6389304 10.1002/hep.1840040625

[27] Sato KK, Hayashi T, Nakamura Y, (2008) Liver enzymes compared with alcohol consumption in predicting the risk of type 2 diabetes: the Kansai Healthcare Study. Diabetes Care 31, 1230–1236. 18316395 10.2337/dc07-2184

[28] Miller PM, Anton RF, Egan BM, (2005) Excessive alcohol consumption and hypertension: clinical implications of current research. J Clin Hypertens (Greenwich) 7, 346–351. 16088298 10.1111/j.1524-6175.2004.04463.x PMC8109365

[29] Lee DH, Ha MH, Kim JH, (2003) γ-Glutamyltransferase and diabetes – a 4 year follow-up study. Diabetologia 46, 359–364. 12687334 10.1007/s00125-003-1036-5

[30] Lee DH, Silventoinen K, Jacobs DR, (2004) γ-Glutamyltransferase, obesity, and the risk of type 2 diabetes: observational cohort study among 20,158 middle-aged men and women. J Clin Endocrinol Metab 89, 5410–5414. 15531490 10.1210/jc.2004-0505

[31] Fraser A, Harris R, Sattar N, (2007) γ-Glutamyltransferase is associated with incident vascular events independently of alcohol intake: analysis of the British Women's Heart and Health Study and Meta-Analysis. Arterioscler Thromb Vasc Biol 27, 2729–2735. 17932318 10.1161/ATVBAHA.107.152298

[32] Aller R, De Luis DA, Izaola O, (2011) Effect of a probiotic on liver aminotransferases in nonalcoholic fatty liver disease patients: a double blind randomized clinical trial. Eur Rev Med Pharmacol Sci 15, 1090–1095. 22013734

[33] Kirpich IA, Solovieva NV, Leikhter SN, (2008) Probiotics restore bowel flora and improve liver enzymes in human alcohol-induced liver injury: a pilot study. Alcohol 42, 675–682. 19038698 10.1016/j.alcohol.2008.08.006 PMC2630703

[34] Kondo S, Kamei A, Xiao JZ, (2013) *Bifidobacterium breve* B-3 exerts metabolic syndrome-suppressing effects in the liver of diet-induced obese mice: a DNA microarray analysis. Benef Microbes 4, 247–251. 23666099 10.3920/BM2012.0019

[35] Baker RG, Hayden MS & Ghosh S (2011) NF-κB, inflammation, and metabolic disease. Cell Metab 13, 11–22. 21195345 10.1016/j.cmet.2010.12.008 PMC3040418

[36] Schenk S, Saberi M & Olefsky JM (2008) Insulin sensitivity: modulation by nutrients and inflammation. J Clin Invest 118, 2992–3002. 18769626 10.1172/JCI34260 PMC2522344

[37] Hotamisligil GS & Erbay E (2008) Nutrient sensing and inflammation in metabolic diseases. Nat Rev Immunol 8, 923–934. 19029988 10.1038/nri2449 PMC2814543

[38] Rocha VZ & Folco EJ (2011) Inflammatory concepts of obesity. Int J Inflam 2011, 529061. 21837268 10.4061/2011/529061 PMC3151511

[39] Weisberg SP, McCann D, Desai M, (2003) Obesity is associated with macrophage accumulation in adipose tissue. J Clin Invest 112, 1796–1808. 14679176 10.1172/JCI19246 PMC296995

[40] Suganami T & Ogawa Y (2010) Adipose tissue macrophages: their role in adipose tissue remodeling. J Leukoc Biol 88, 33–39. 20360405 10.1189/jlb.0210072

[41] Suganami T, Nishida J & Ogawa Y (2005) A paracrine loop between adipocytes and macrophages aggravates inflammatory changes: role of free fatty acids and tumor necrosis factor α. Arterioscler Thromb Vasc Biol 25, 2062–2068. 16123319 10.1161/01.ATV.0000183883.72263.13

[42] Cani PD, Bibiloni R, Knauf C, (2008) Changes in gut microbiota control metabolic endotoxemia-induced inflammation in high-fat diet-induced obesity and diabetes in mice. Diabetes 57, 1470–1481. 18305141 10.2337/db07-1403

[43] Cani PD, Possemiers S, Van de Wiele T, (2009) Changes in gut microbiota control inflammation in obese mice through a mechanism involving GLP-2-driven improvement of gut permeability. Gut 58, 1091–1103. 19240062 10.1136/gut.2008.165886 PMC2702831

[44] Hoarau C, Lagaraine C, Martin L, (2006) Supernatant of *Bifidobacterium breve* induces dendritic cell maturation, activation, and survival through a Toll-like receptor 2 pathway. J Allergy Clin Immunol 117, 696–702. 16522473 10.1016/j.jaci.2005.10.043

[45] Bermudez-Brito M, Muñoz-Quezada S, Gomez-Llorente C, (2013) Cell-free culture supernatant of *Bifidobacterium breve* CNCM I-4035 decreases pro-inflammatory cytokines in human dendritic cells challenged with *Salmonella typhi* through TLR activation. PLOS ONE 8, e17. 10.1371/journal.pone.0059370 PMC3595273 23555025

[46] Jeon SG, Kayama H, Ueda Y, (2012) Probiotic *Bifidobacterium breve* induces IL-10-producing Tr1 cells in the colon. PLoS Pathog 8, e17. 10.1371/journal.ppat.1002714 PMC3364948 22693446

